# A retrospective analysis of the care cascades for non‐communicable disease and mental health among people living with HIV at a tertiary‐care centre in Malaysia: opportunities to identify gaps and optimize care

**DOI:** 10.1002/jia2.25638

**Published:** 2020-11-18

**Authors:** Chong Meng Li, Fong Jie Ying, Dhevann Raj, Wong Pui Li, Anjanna Kukreja, Sharifah FS Omar, Adeeba Kamarulzaman, Reena Rajasuriar

**Affiliations:** ^1^ Centre of Excellence for Research in AIDS (CERiA) University of Malaya Kuala Lumpur Malaysia; ^2^ Department of Pharmacy University of Malaya Kuala Lumpur Malaysia; ^3^ Department of Medicine University of Malaya Kuala Lumpur Malaysia

**Keywords:** chronic disease, mental health, morbidity, elderly, linkage to care, health services, HIV

## Abstract

**Introduction:**

The rapidly growing epidemic of non‐communicable diseases (NCDs) including mental health among aging people living with HIV (PLWH) has put a significant strain on the provision of health services in many HIV clinics globally. We constructed care cascades for specific NCDs and mental health among PLWH attending our centre to identify potential areas for programmatic improvement.

**Methods:**

This was a follow‐up study of participants recruited in the Malaysian HIV & Aging study (MHIVA) from 2014 to 2016 at the University Malaya Medical Centre (n = 336). PLWH on suppressive antiretroviral therapy (ART) for a minimum of 12 months were invited to participate. At study entry, all participants underwent screening for diabetes (DM), hypertension (HTN) and dyslipidaemia; and completed assessments using the depression, anxiety and stress scale (DASS‐21). Screening results were recorded in medical charts and clinical management provided as per standard of care. A subsequent review of medical records was performed at 24 months following study completion among participants who remained on active follow‐up. Treatment pathways for NCD treatment and psychiatric referrals were assessed based on local practice guidelines to construct the care cascade.

**Results:**

A total of 329 participants (median age = 43 years, 83% male, 100% on ART) completed follow‐up at 24 months. The prevalence of diabetes was 13%, dyslipidaemia 88% and hypertension 44%, whereas 23% presented with severe/extremely severe symptoms of depression, anxiety and/or stress. More than 50% of participants with dyslipidaemia and hypertension were not diagnosed until study screening, whereas over 80% with prevalent psychiatric symptoms were not previously recognized clinically. Suboptimal control of fasting lipids, sugar and blood pressure were found in the majority of participants despite optimal HIV treatment outcomes maintained over this same period. Only 32% of participants with severe/extremely severe mental health symptoms received psychiatric referrals and 83% of these attended their psychiatry clinic appointments.

**Conclusions:**

Systematic screening must be introduced to identify NCDs and mental health issues among PLWH followed by proper linkage and referrals for management of screen‐positive cases. Assessment of factors associated with attrition at each step of the care cascade is critically needed to improve health outcomes in our aging patients.

## 1 INTRODUCTION

The introduction of antiretroviral therapy has progressively improved the life expectancy of people living with HIV (PLWH) [1]. Consequently, the global demographics of PLWH has changed with approximately 7.5 million individuals over the age of 50 years [2]. In the Asia‐Pacific region, one in three PLWH are expected to be over the age of 50 years by 2025 [3]. This age advancement is associated with an increased prevalence of chronic comorbidities [4] and geriatric syndromes [5, 6] among PLWH, leading to calls for HIV treatment programmes to adapt a multi‐disciplinary team approach to better address the healthcare needs of the population [7].

Among the Asia‐Pacific LMIC countries including Malaysia, the care of PLWH is still largely centred in tertiary care settings and in many instances, HIV specialists are the sole healthcare provider for PLWH. HIV doctors thus inadvertently carry the additional responsibility of primary care in screening for non‐communicable diseases, mental health and cancer [8]. Furthermore, high clinic case loads, limited support personnel and poor integration between specialty care services make it challenging for HIV treatment programmes in the region to adopt the call to establish multidisciplinary care models to manage our patients as they age. Though various models of integrated care for PLWH have been introduced, these have largely been in resource‐rich settings and evolved from well established and better resourced health systems compared to those typically found in the LMIC setting [7].

At our treatment centre, we previously identified a high burden of geriatric conditions among PLWH on stable ART on routine follow‐up which was associated with poorer quality of life, higher mortality risks and increased healthcare utilization [5]. This study highlighted an urgent need to expand our HIV care to beyond that of virological and immunological outcomes. To this end, we constructed care cascades for the management of specific non‐communicable diseases including mental health among PLWH on routine follow‐up in our clinic to identify key areas in care provision which needed improvement. This pre‐implementation assessment has highlighted important constructs at the patient, provider, organizational and process levels which need to be addressed when considering strategies to integrate NCD and mental healthcare into HIV services in our setting.

## 2 METHODS

This was a follow‐up study of participants from the Malaysian HIV and Aging (MHIVA) cohort where participants under routine care at the Infectious Diseases Unit, University Malaya Medical Centre (UMMC) were recruited between October 2014 and March 2016. The inclusion criteria for the study was age ≥25 years, HIV RNA < 50 copies/mL on antiretroviral therapy (ART) for at least 12 months and no acute illness on recruitment.

Details of the study and cohort characteristics (N = 336) have been previously described [5]. Briefly, all participants underwent assessments for chronic comorbidities including screening for symptoms of depression, anxiety and stress as well as dyslipidaemia, hypertension and diabetes. Abnormal results from screening were reviewed by study doctors and noted in participants case notes to be addressed at their next clinic visit (within three to six months) or sooner if deemed urgent. Clinical management of abnormal screens were done as per standard of care and described in Table 1.

**Table 1:** Definitions used to determine abnormal screening results, target outcomes following treatment and treatment pathways for each NCD

NCDs	Definition of abnormal screening and indication for treatment*/referral	Target outcome following treatment intervention	Treatment setting and pathway
Dyslipidaemia	LDL‐C ≥ 3.4mmol/L TC ≥ 5.2mmol/L HDL‐C < 1.0mmol/L TG ≥ 1.7mmol/L	CVD and CHD risk equivalents (High risk^#^) LDL‐C < 2.0 (very high risk) or 2.6 mmol/L Individuals with a 10‐year risk score of 10–20% (Intermediate risk^#^) LDL‐C < 3.4 mmol/L Individuals with a 10‐year risk score of < 10% (Low risk^#^) LDL‐C < 4.1 mmol/L	Treatment by HIV physicians during routine HIV clinic follow‐up
Hypertension	BP ≥ 140/90 mmHg (Stage 1 HPT) BP ≥ 160/100 mmHg (Stage 2 HPT) BP ≥ 180/110 mmHg (Severe HPT)	Age < 80 years with treated hypertension < 140/90 mmHg Age ≥ 80 years with treated hypertension < 150/90 mmHg High/very high‐risk patient < 130 or 140/80 mmHg	Treatment by HIV physicians during routine HIV clinic follow‐up
Diabetes Mellitus	FBG ≥ 7mmol/L HbA1c ≥ 6.5%	FBG: 4.4‐6.1 mmol/L HbA1c: <6.5 %	Treatment by HIV physicians during routine HIV clinic follow‐up
Mental Health (Depression, anxiety and stress)	Referral to psychologist or psychiatry clinic if scores ≥ 11 (depression) ≥8 (anxiety) and ≥ 13 (stress) on DASS‐21 indicating severe or extremely severe symptoms.	–	Treatment by psychologist or psychiatrist at the out‐patient psychiatry clinic within the same facility with referrals or as walk‐ins

BP, blood pressure; CHD, coronary heart diseases; CVD, cardiovascular diseases; DM, diabetes mellitus; FBG, fasting blood glucose; HbA1c, glycosylated haemoglobin A protein test; HDL, high‐density lipoprotein; HPT, hypertension; LDL, low‐density lipoprotein; TC, total cholesterol; TG, triglyceride.

^a^ Framingham CHD Risk category; *includes lifestyle modification and/or drug intervention.

In this study, a review of medical records was performed of participants who remained on active follow‐up in our clinic (n = 329) to construct care cascades for NCD and mental health. Participants provided written informed consent for the study team to access medical records up to five years following recruitment. The study protocol was approved by the institutional review board (MEC 20151‐937).

### 2.1 Construction of care cascade for non‐communicable diseases

At recruitment into MHIVA, all participants had fasting bloods taken to assess lipids, glucose and HbA1c while serial blood pressures were done to assess for hypertension. Prior diagnosis of NCDs were ascertained from multiple sources; review of medical records, pharmacy records and self‐reports by participants. The prevalence of each NCD was calculated from both the number of new diagnosis identified as a result of study screening as well as from previous diagnosis. Records of participants with a new or previous NCD diagnosis were reviewed at 24 months following recruitment and assessed for receipt of treatment; defined as having received either advice on lifestyle modification or medication at any point during follow‐up. Control of abnormal lab values to recommended target thresholds were also assessed at 24 months and defined based on local clinical practice guidelines [9, 10, 11] and summarized in Table 1.

### 2.2 Construction of care cascade for mental health

All participants were also screened for depression, anxiety and stress at recruitment with the DASS‐21 questionnaire while prior mental health diagnosis were assessed from medical and pharmacy records. Assessment of participants with prevalent psychiatric symptoms requiring referrals are defined in Table 1. At 24 months of follow‐up, medical records of those with prevalent symptoms were assessed for formal referrals by the HIV doctor to the psychiatric unit and the participants follow‐up attendance at the psychiatric clinic.

We additionally explored the care cascade for HIV management in the same period. Indicators assessed were the proportion of participants on follow‐up who were still receiving antiretroviral therapy, maintained HIV RNA levels at <50 copies/mL and had >80% compliance of HIV clinic attendance over the follow‐up period. A missed appointment or a change in appointment dates beyond three months of the original date were assessed as non‐compliant.

## 3 RESULTS AND DISCUSSION

A total of 329 from the 336 participants initially recruited in the MHIVA cohort remained on active follow‐up at our unit after 24 months. The majority of participants were males (82.7%) and the median age was 44 (interquartile range, IQR 38‐51) years. Participants had received ART for a median duration of 7 (4‐11) years at the point of study inclusion. Baseline CD4 T cell count was 110 (35‐246) cells/µL, whereas current CD4 T‐cell count was 571 (403‐730) cells/µL. The majority received efavirenz (NNRTI)‐based regimen (85%) followed by a PI‐based regimen (12%).

**Figure 1:**
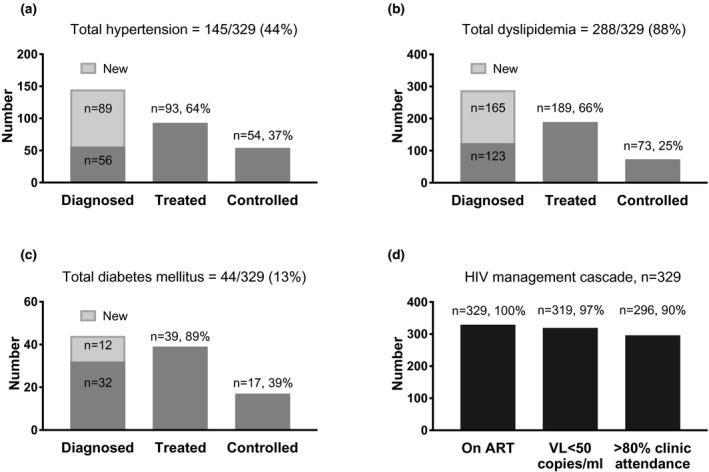
Treatment cascades for non‐communicable diseases and HIV disease among PLWH (n = 329). Non‐communicable disease treatment cascades (grey bars) were constructed for (A) hypertension, (B) dyslipidaemia and (C) diabetes mellitus from laboratory and clinical data collected following screening of the Malaysian HIV and Aging study participants until 24 months after the end of study recruitment. Participants with a diagnosis (new or previous) who received either lifestyle modification advice or medication by the end of the assessment period were considered as treated. Participants who achieved target thresholds for HbA1C or fasting blood sugar (diabetes), LDL‐cholesterol (dyslipidaemia) and systolic blood pressure (hypertension), were considered as controlled. Care cascades for (D) HIV management (black bars) were also constructed for HIV‐related indicators assessed over the same period.

The prevalence of NCDs were high and similar to other cohorts of PLWH in the region [12, 13, 14], with diabetes found in 13%, dyslipidaemia 88% and hypertension 44%. For all three NCDs assessed, a significant proportion of participants were previously undiagnosed and identified only as a result of study screening, implying a poor practice of screening for NCDs as part of routine HIV care (Figure 1). This observation has also been documented in other HIV clinic cohorts in the region [15, 16]. By 24 months of follow‐up, 89% of those diagnosed with diabetes (n = 44) had received treatment (16% lifestyle only, 84% medication). However, fewer participants with a diagnosis of dyslipidaemia and hypertension were on treatment, 66% and 64%, respectively, and this could be associated with the asymptomatic nature of these conditions. Thirty‐three percent of participants treated for dyslipidaemia and 23% treated for hypertension were managed with lifestyle modifications alone (results not shown). The proportion of participants achieving target laboratory outcomes for NCDs were also suboptimal in our cohort with control of lipid levels being the poorest (25% of all diagnosed).

**Figure 2:**
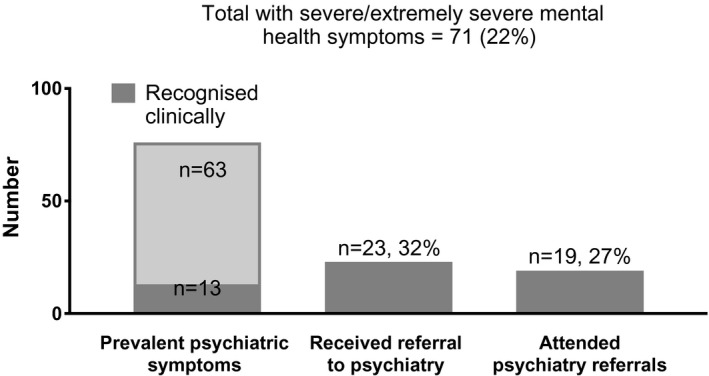
Abbreviated treatment cascade for PLWH presenting with extreme to extremely severe symptoms of depression, anxiety and/or stress requiring psychiatric referrals (n = 329). The care cascade for mental health issues were assessed from screening conducted as part of the Malaysian HIV and Aging (MHIVA) study to the first presentation to a psychiatrist/psychologist within 24 months after the end of study recruitment. New diagnosis with prevalent mental health symptoms requiring referrals were defined as individuals with scores ranging from severe to extremely severe on the Depression, Anxiety and Stress questionnaire (DASS‐21) Individuals with symptoms and a prior mental health diagnosis are indicated as ‘recognized clinically’. The proportion of those with prevalent symptoms who subsequently received referrals to a psychiatrist or psychologist by 24 months following end of recruitment were noted as were those who then followed through with their first appointment.

We documented the care cascade for HIV treatment (Figure 2) to explore if poor NCD management over the 24‐month period could have been contributed by poor participant attendance and thus the lack of opportunity to optimize NCD care. However, we did not find this to be a potential reason for the observed results as prevalence for all HIV care indicators were at least 90%. Over the 24‐month observation period, participants appeared to have well‐controlled HIV disease, but a large proportion experienced undiagnosed and/or sub‐optimal control of hypertension, dyslipidaemia and diabetes. Poor control of NCDs has recently been described to be a significant contributor of fatal and non‐fatal cardiovascular events among PLWH on ART in the Asia‐Pacific region [17, 18] and addressing this gap should be made a programmatic priority in our clinic.

Participants in this study, by nature of their inclusion criteria, were PLWH with no outstanding HIV‐related medical issues and on long‐term ART. The latter, is in itself associated with metabolic and CVD complications [19] but often overlooked. In our setting, patients of this profile tend to be seen by trainee/junior doctors who may not be familiar with the need or importance of screening for NCDs. We speculate that this oversight may have contributed to the poor management of NCDs among PLWH in our setting despite their HIV care being optimal over the same period. Additionally, as clinic loads are high, patients in queue tend to be seen by any available doctor and this system is not conducive in ensuring care plans are continued especially in the context of Malaysia where shared‐decision making is not widely practiced [20, 21]. This could potentially have contributed to participants remaining on lifestyle modification as their primary treatment for an extended period despite not achieving optimal NCD control. We also could not rule out the reluctance of participants to initiate medications for NCD due to concerns of pill burden and side effects despite being advised by their doctors (personal communication). Additionally, ART medications are government subsidized, whereas NCD medications are not. Our findings collectively imply a poor appreciation by both HIV providers and patients of the importance of optimizing care for NCDs. Whether this is due to inadequate training among providers, poor awareness among both providers and patients, gaps in health financing or other structural/process failures needs to be explored further.

The treatment cascade for PLWH presenting with mental health symptoms/diagnosis showed greater gaps in screening and management. Only 18% (n = 13) of participants with severe or extremely severe symptoms of depression (n = 33), anxiety (n = 18) and/or stress (n = 50) (many with overlapping conditions) (n = 71, 22%) were recognized clinically and had a previous mental health diagnosis (Figure 2). This is consistent with the lack of routine mental health screening practice in our HIV clinic. To our surprise, only 32% of participants requiring further psychiatric assessment following screening with DASS‐21 received referrals to see a psychiatrist/psychologist. Of note and contrary to prior reports of significant drop outs in clinic attendance for mental health services following referrals [22, 23], we found that the majority of participants who received referrals to the psychiatric clinic met their appointments.

The management of mental health among participants showed a clear lapse in screening practices and recognition of the need for referrals by HIV care providers. Screening for mental health is not routinely performed in many HIV treatment sites in the Asia‐Pacific region [8, 24] despite high reported prevalence for a range of mental health disorders among PLWH [25, 26, 27]. The sentiment and clinical experience of our HIV providers are that participants are reluctant to attend psychiatry clinics likely due to stigma (personal communication) and thus in the absence of opportunities for intervention, there is limited value to perform screening. Prior efforts to organize joint HIV and mental health/substance abuse clinics in our unit have not materialized due to staff shortages and poor coordination. Our assessment of the treatment cascade, however, challenges this sentiment as we found that the majority of participants who received referrals did attend appointments at the psychiatry clinic. However, our study did not include an assessment of the adequacy of treatment obtained via this care pathway which could take considerably longer than 24 months. Future studies will need to thoroughly explore reasons for the attrition along the mental healthcare cascade including effectiveness of treatment and the need for additional training in mental health assessment for HIV providers, their readiness and barriers to providing referrals and patients stigma experience in accessing mental healthcare services.

## 4 CONCLUSIONS

In conclusion, we identified a high rate of NCDs and mental health issues requiring follow‐up care among PLWH attending our HIV clinic. Assessment of the care cascade for these conditions have identified key areas which need to be urgently addressed; specifically improving screening practices and linkage to care, as well as optimizing treatment outcomes. Future studies will need to systematically assess factors which facilitate or impede these processes within our HIV treatment programme in order to improve health outcomes in our patients as they age.

## COMPETING INTERESTS

The authors have no conflict of interest to declare.

## AUTHORS’ CONTRIBUTIONS

RR, AK, SFSO and AK conceived the study; CML, DR, WPL, AK and FJY contributed to recruitment and data collection, DR, FJY, CML, WPL and RR analysed the data; RR and CML wrote and formatted the manuscript; RR, SFSO and AK contributed to funding. All authors reviewed and approved the final manuscript.
